# Supplementary material for: A Follow up on the Continuum Theory of Eco-Anxiety: Analysis of the Climate Change Anxiety Scale Using Item Response Theory among French Speaking Population
Source: Int J Environ Res Public Health. 2024 Aug 30;21(9):1158. doi: 10.3390/ijerph21091158 (PMC11431234; doi:10.3390/ijerph21091158)
Supplement: Supplementary file 1 [file ijerph-21-01158-s001.zip › ijerph-3121223-supplementary.docx]

**Supplementary Materials:**

**S1 : Climate Change Anxiety Scale — short versionvalidated in French by Mouguiama-Daouda et al. (2022) :**

Please indicate how well each of the following statements applies to you. There are no right or wrong answers, so answer with your first intuition and don't dwell too long on the statements. [*Veuillez indiquer à quel point chacun des énoncés suivants vous correspondent. Il n'y a pas de bonnes ou de mauvaises réponses, répondez avec votre première intuition et ne vous attardez pas trop longuement sur les énoncés.*]

1. Thinking about climate change makes it difficult for me to concentrate.[*Il m’est difficile de me concentrer lorsque je pense au changement climatique.*]
2. Thinking about climate change makes it difficult for me to sleep. [*Il m’est difficile de m’endormir lorsque je pense au changement climatique.*]
3. I have nightmares about climate change. [*Je fais des cauchemars à propos du changement climatique.*]
4. I find myself crying because of climate change. [*Je me retrouve à pleurer à cause du changement climatique.*]
5. I think, “why can't I handle climate change better? [*Je me demande pourquoi est-ce que je n’arrive pas à mieux gérer le changement climatique.*]
6. I go away by myself and think about why I feel this way about climate change. [*Je me mets à l'écart et pense aux raisons pour lesquelles je me sens ainsi face au changement climatique.*]
7. I write down my thoughts about climate change and analyze them. [*J'écris mes réflexions sur le changement climatique et je les analyse.*]
8. I think, “why do I react to climate change this way?”[Je me demande pourquoi je réagis ainsi au changement climatique.]
9. My concerns about climate change make it hard for me to have fun with my family or friends. [*Mes préoccupations au sujet du changement climatique m'empêchent de m'amuser avec ma famille ou mes amis.*]
10. I have problems balancing my concerns about sustainability with the needs of my family. [*J’ai du mal à trouver un équilibre entre mes préoccupations pour le « durable » et les besoins de ma famille.*]
11. My concerns about climate change interfere with my ability to get work or school assignments done. [*Mes préoccupations à propos du changement climatique interfèrent avec ma capacité à faire mon travail ou mes tâches scolaires.*]
12. My concerns about climate change undermine my ability to work to my potential. [*Mes préoccupations concernant le changement climatique impactent négativement ma capacité à exploiter pleinement mon potentiel.*]
13. My friends say I think about climate change too much. [*Mes amis disent que je pense trop au changement climatique.*]

Respondents answer on a 5 points Likert scale : 1 : Never, 2 : Rarely, 3 : Sometimes, 4 : Often, 5 : Almost always

**S2 : Model fit and psychometric quality analyses of CCAS-13 using the second data set (N=873)**

**Model comparison of the second data set (N=873)**

| Model | *M*_2_ | df | *p* | RMSEA | SRMSR | TLI | CFI | AIC |
| --- | --- | --- | --- | --- | --- | --- | --- | --- |
| 1 factor | 334.05 | 26 | <.001 | 0.117 | 0.072 | 0.78 | 0.86 | 26497.48 |
| 2 factors | 211.58 | 25 | <.001 | 0.093 | 0.068 | 0.87 | 0.91 | 26364.65 |
| 3 factors  (Exploratory) | 0.52 | 3 | 0.914 | 0 | 0.046 | 1 | 1 | 26061.69 |
| 3 factors (confirmatory) | 65.56 | 23 | <.001 | 0.046 | 0.046 | 0.97 | 0.97 | 26087.81 |
| bifactor | 34.61 | 13 | <.001 | 0.043 | 0.05 | 0.97 | 0.99 | 26038.39 |

*Note*. χ2 : Pearson’s test statistic, *M_2_* : the limited information goodness-of-fit test statistic, df: degree of freedom, *p* : p value, RMSEA : Root Mean Square Error Approximation, SRMSR: Standardized Root Mean Squared Residual, TLI: Tucker-Lewis Index, CFI: Comparative Fit Index, AIC: Akaike Information Criterion

**Comparison between the 1 to 2 and 3 dimensional models and the bifactor model**

|  | AIC | χ^2^ | df | *p* |
| --- | --- | --- | --- | --- |
| Modèle à 1 facteurs | 26497.48 |  |  |  |
| Modèle à 2 facteurs | 26364.65 | 134.833 | 1 | <.001 |
| Modèle à 3 facteurs (confirmatory) | 26087.81 | 280.835 | 2 | <.001 |
| Modèle bifactoriel | 26038.39 | 69.424 | 10 | <.001 |

*Note*. AIC: Akaike Information Criterion, χ2: Pearson’s test statistic, df: degree of freedom, *p*: p value

**Exploratory 3-factor model without rotation (N=873)**

Item saturation :

|  | F1 | F2 | F3 |
| --- | --- | --- | --- |
| CCAS1 | -0.652 | 0.1598 | -0.4646 |
| CCAS2 | -0.677 | 0.3146 | -0.5972 |
| CCAS3 | -0.605 | 0.0747 | -0.3986 |
| CCAS4 | -0.620 | 0.0504 | -0.3856 |
| CCAS5 | -0.613 | -0.4015 | -0.2927 |
| CCAS6 | -0.736 | -0.3521 | -0.2680 |
| CCAS7 | -0.390 | -0.1534 | -0.0972 |
| CCAS8 | -0.522 | -0.5022 | -0.2078 |
| CCAS9 | -0.818 | 0.0570 | -0.0824 |
| CCAS10 | -0.624 | -0.0223 | -0.0416 |
| CCAS11 | -0.857 | 0.0385 | 0.0449 |
| CCAS12 | -0.855 | 0.0638 | 0.0000 |
| CCAS13 | -0.624 | 0.0000 | 0.0000 |

|  | F1 | F2 | F3 |
| --- | --- | --- | --- |
| Variance expliquée | 0.453 | 0.054 | 0.085 |

**Exploratory 3-factor model with Oblimin rotation (N=873)**

Item saturation:

|  | F1 | F2 | F3 |
| --- | --- | --- | --- |
| CCAS1 | 0.0630 | 0.0777 | 0.7186 |
| CCAS2 | -0.0203 | -0.0587 | 1.0037 |
| CCAS3 | 0.0667 | 0.1538 | 0.5720 |
| CCAS4 | 0.0881 | 0.1807 | 0.5372 |
| CCAS5 | -0.0144 | 0.7384 | 0.0988 |
| CCAS6 | 0.1631 | 0.6681 | 0.0966 |
| CCAS7 | 0.1652 | 0.2858 | 0.0140 |
| CCAS8 | -0.0347 | 0.8223 | -0.0828 |
| CCAS9 | 0.7042 | 0.0267 | 0.1285 |
| CCAS10 | 0.5343 | 0.0975 | 0.0237 |
| CCAS11 | 0.9094 | -0.0128 | -0.0566 |
| CCAS12 | 0.8576 | -0.0232 | 0.0214 |
| CCAS13 | 0.6033 | 0.0457 | -0.0167 |

Factor correlations

|  | F1 | F2 | F3 |
| --- | --- | --- | --- |
| F1 | 1.000 |  |  |
| F2 | 0.718 | 1.000 |  |
| F3 | 0.748 | 0.585 | 1 |

**3-factor model (confirmatory analysis):**

Item saturation

|  | SYM | RUM | FI |
| --- | --- | --- | --- |
| CCAS1 | 0.818 |  |  |
| CCAS2 | 0.895 |  |  |
| CCAS3 | 0.754 |  |  |
| CCAS4 | 0.742 |  |  |
| CCAS5 |  | 0.765 |  |
| CCAS6 |  | 0.885 |  |
| CCAS7 |  | 0.440 |  |
| CCAS8 |  | 0.681 |  |
| CCAS9 |  |  | 0.829 |
| CCAS10 |  |  | 0.631 |
| CCAS11 |  |  | 0.841 |
| CCAS12 |  |  | 0.850 |
| CCAS13 |  |  | 0.625 |

|  | SYM | RUM | FI |
| --- | --- | --- | --- |
| Explained variance | 0.199 | 0.156 | 0.224 |

Factor correlation

|  | SYM | RUM | FI |
| --- | --- | --- | --- |
| SYM | 1.000 |  |  |
| RUM | 0.691 | 1.000 |  |
| FI | 0.800 | 0.786 | 1 |

*Note*. SYM: Physiological symptoms, RUM: Rumination, FI: functional impairment

**Confirmatory 2-factor model:**

Saturation des items

|  | CEI | FI |
| --- | --- | --- |
| CCAS1 | 0.784 | 0.000 |
| CCAS2 | 0.820 | 0.000 |
| CCAS3 | 0.739 | 0.000 |
| CCAS4 | 0.749 | 0.000 |
| CCAS5 | 0.687 | 0.000 |
| CCAS6 | 0.787 | 0.000 |
| CCAS7 | 0.409 | 0.000 |
| CCAS8 | 0.567 | 0.000 |
| CCAS9 | 0.000 | 0.841 |
| CCAS10 | 0.000 | 0.640 |
| CCAS11 | 0.000 | 0.848 |
| CCAS12 | 0.000 | 0.858 |
| CCAS13 | 0.000 | 0.638 |

|  | CEI | FI |
| --- | --- | --- |
| Explained variance | 0.306 | 0.229 |

Factor correlation

|  | CEI | FI |
| --- | --- | --- |
| CEI | 1.000 |  |
| FI | 0.879 | 1 |

*Note*. CEI: cognitive-emotional impairment, FI: functional impairment

**Bifactor Model (N=873):**

Saturation des items :

|  | G | S1 | S2 |
| --- | --- | --- | --- |
| CCAS1 | 0.767 | 0.291 |  |
| CCAS2 | 0.827 | 0.463 |  |
| CCAS3 | 0.712 | 0.188 |  |
| CCAS4 | 0.724 | 0.157 |  |
| CCAS5 | 0.718 | -0.273 |  |
| CCAS6 | 0.832 | -0.290 |  |
| CCAS7 | 0.427 | -0.150 |  |
| CCAS8 | 0.612 | -0.396 |  |
| CCAS9 | 0.749 |  | 0.338 |
| CCAS10 | 0.558 |  | 0.303 |
| CCAS11 | 0.715 |  | 0.501 |
| CCAS12 | 0.729 |  | 0.485 |
| CCAS13 | 0.606 |  | 0.161 |

|  | G | S1 | S2 |
| --- | --- | --- | --- |
| Explained variance | 0.489 | 0.054 | 0.055 |

*Note*. G: general factor measuring eco-anxiety, S1: first subdomain representing cognitive-emotional impairment, S2: second subdomain representing functional impairment

**Unidimensional model (N = 873):**

Saturation des items

|  | Eco-anxiety |
| --- | --- |
| CCAS1 | 0.752 |
| CCAS2 | 0.789 |
| CCAS3 | 0.702 |
| CCAS4 | 0.716 |
| CCAS5 | 0.679 |
| CCAS6 | 0.785 |
| CCAS7 | 0.408 |
| CCAS8 | 0.561 |
| CCAS9 | 0.813 |
| CCAS10 | 0.615 |
| CCAS11 | 0.804 |
| CCAS12 | 0.818 |
| CCAS13 | 0.636 |

|  | Eco-anxiety |
| --- | --- |
| Explained variance | 0.501 |

**Psychometric qualities of CCAS-13 for the second data set (N=873)**

**Infit/ outfit parameters**

| item | outfit | infit |
| --- | --- | --- |
| CCAS1 | 0.871 | 0.910 |
| CCAS2 | 0.824 | 0.894 |
| CCAS3 | 0.815 | 0.956 |
| CCAS4 | 0.796 | 0.933 |
| CCAS5 | 0.921 | 0.936 |
| CCAS6 | 0.856 | 0.903 |
| CCAS7 | 1.023 | 0.993 |
| CCAS8 | 0.934 | 0.970 |
| CCAS9 | 0.816 | 0.884 |
| CCAS10 | 0.914 | 0.928 |
| CCAS11 | 0.789 | 0.903 |
| CCAS12 | 0.791 | 0.886 |
| CCAS13 | 0.874 | 0.943 |

**Discrimination parameters *a* and difficulty parameters *b***

|  | *a* | *b_1_* | *b_2_* | *b_3_* | *b_4_* |
| --- | --- | --- | --- | --- | --- |
| CCAS1 | 1,944 | -1,08 | -0,11 | 1,031 | 2,366 |
| CCAS2 | 2,185 | -0,798 | 0,118 | 1,141 | 2,073 |
| CCAS3 | 1,679 | 0,329 | 1,305 | 2,603 | 4,132 |
| CCAS4 | 1,748 | 0,307 | 1,03 | 2,034 | 3,139 |
| CCAS5 | 1,576 | -0,763 | -0,082 | 0,931 | 2,227 |
| CCAS6 | 2,155 | -0,135 | 0,563 | 1,386 | 2,573 |
| CCAS7 | 0,761 | 0,996 | 2,078 | 3,828 | 5,489 |
| CCAS8 | 1,155 | -0,16 | 0,813 | 2,08 | 3,863 |
| CCAS9 | 2,379 | -0,496 | 0,331 | 1,541 | 2,643 |
| CCAS10 | 1,327 | -1,394 | -0,491 | 0,745 | 2,294 |
| CCAS11 | 2,301 | -0,175 | 0,622 | 1,464 | 2,196 |
| CCAS12 | 2,42 | -0,247 | 0,498 | 1,269 | 2,199 |
| CCAS13 | 1,404 | -0,286 | 0,55 | 1,706 | 2,983 |

**The order of items according to their general level of difficulty (ascending order)**

|  | Average item difficulty |
| --- | --- |
| CCAS10 | 0,211 |
| CCAS1 | 0,488 |
| CCAS5 | 0,508 |
| CCAS2 | 0,631 |
| CCAS12 | 0,901 |
| CCAS9 | 0,954 |
| CCAS6 | 1,022 |
| CCAS11 | 1,036 |
| CCAS13 | 1,19 |
| CCAS8 | 1,557 |
| CCAS4 | 1,582 |
| CCAS3 | 2,001 |
| CCAS7 | 3,059 |

**
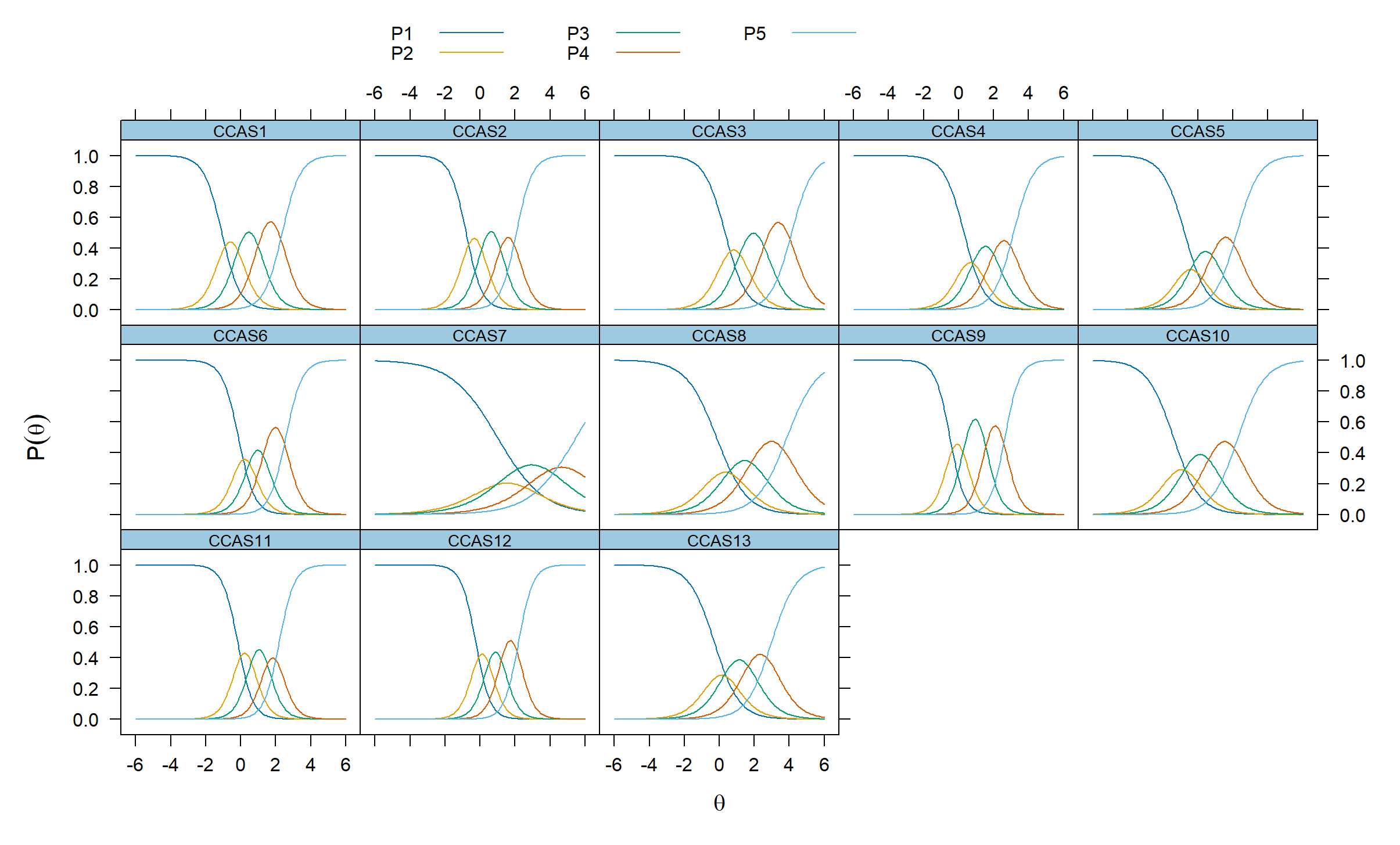
**

Item function curve: P1 to P5: item characteristic curve for the scale categories P1 for “never”, P2 for “rarely”, P3 for “sometimes, P4 for “often”, P5 for “always”; P(θ): probability of selecting a category

**
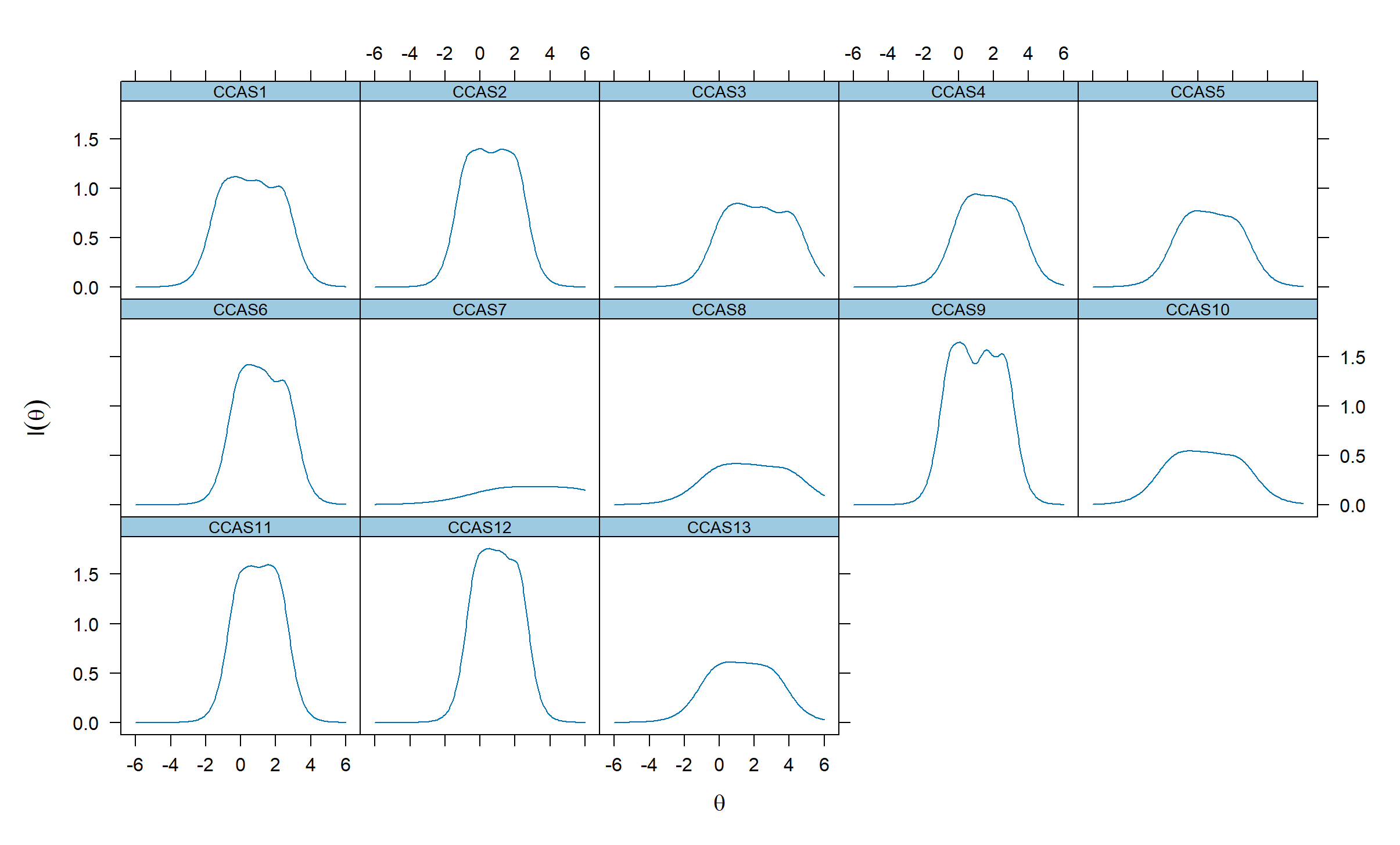
**

Item information curve (N=873): I(θ) quantity of information about the latent trait provided by the item

**
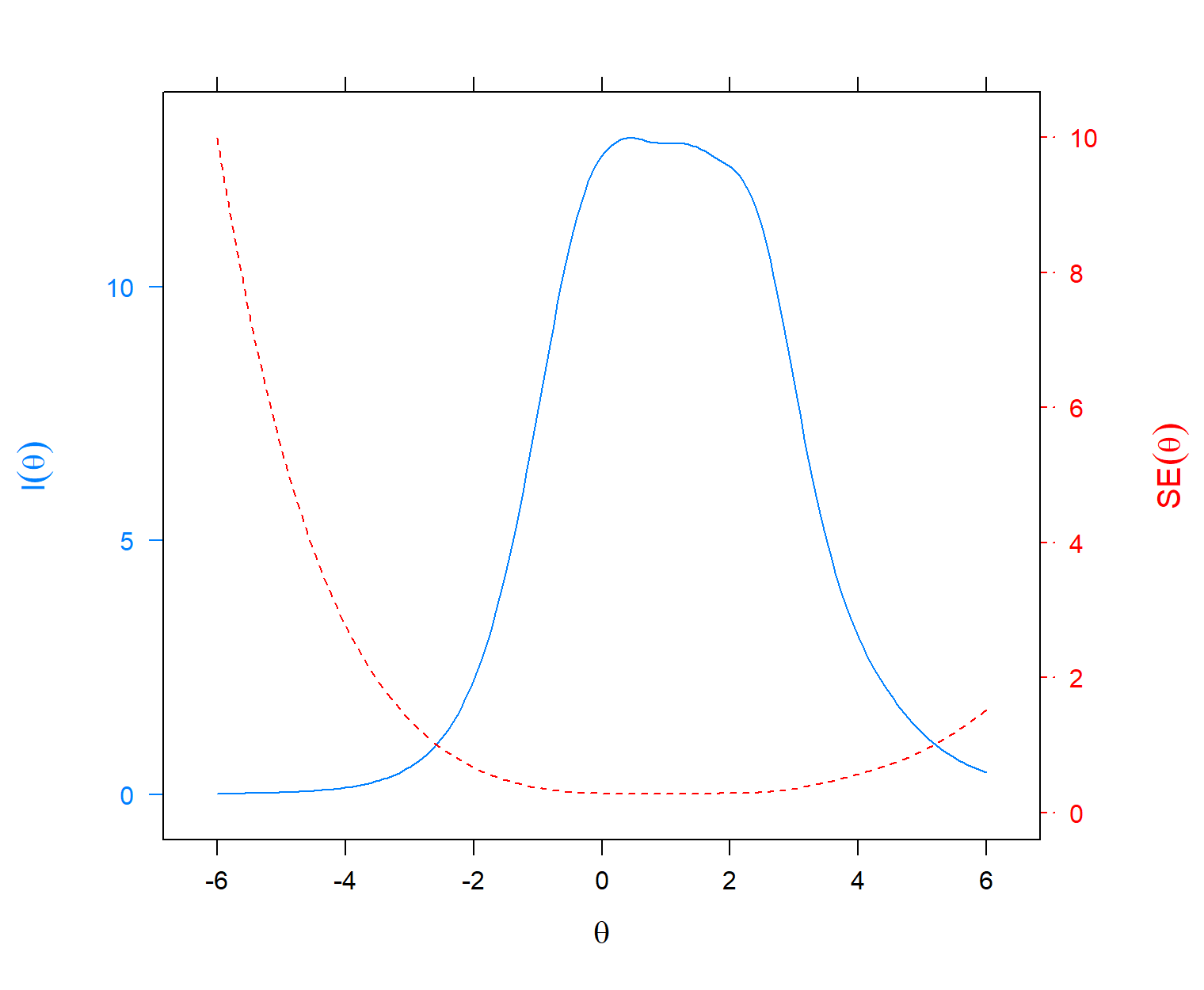
**

Scale information curve: I(θ) quantity of information about the latent trait provided by the whole questionnaire, SE(θ): conditional standard errors indicating how precisely scores can be estimated across different values of the latent trait

**
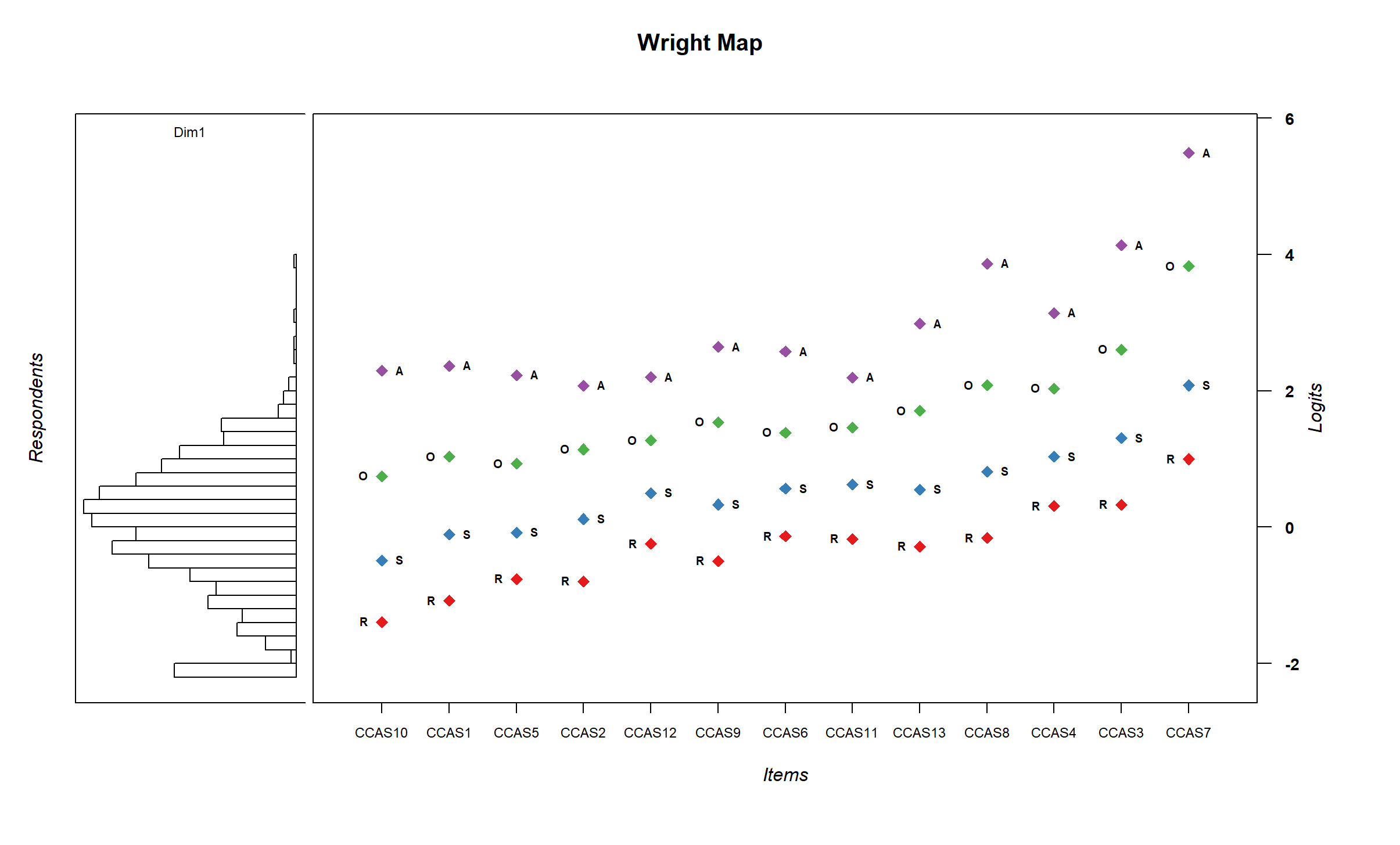
**

Wright map (N=873): right panel represents participants' scores on the eco-anxiety dimension, left panel represents thresholds for having a 50% chance of endorsing a particular modality with A = always, O = often, S= sometimes R = rarely

**
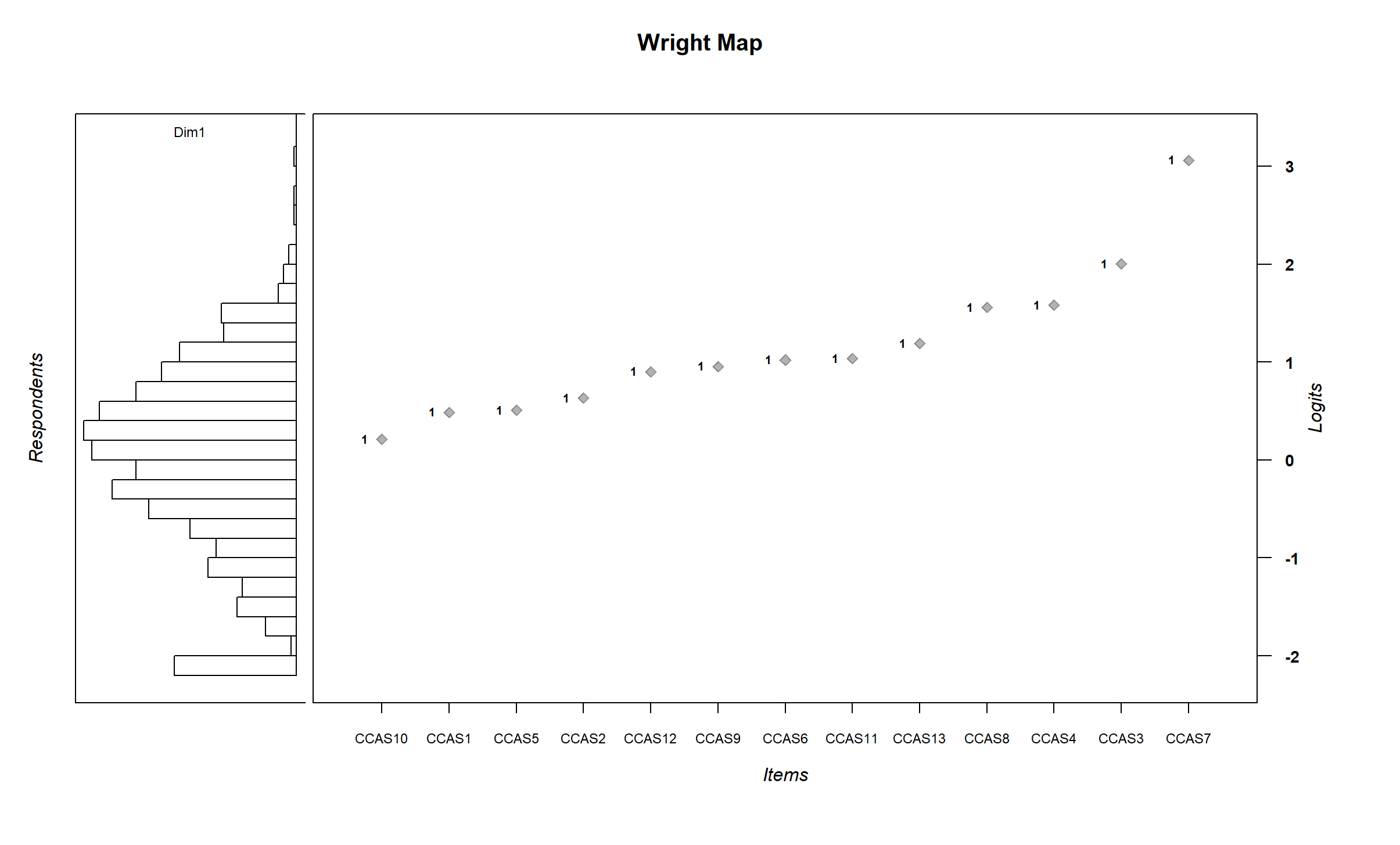
**

Wright map (N =873): right panel represents participants' scores on the eco-anxiety dimension, left panel represents the items classified by their general level of difficulty.

**S3: Supplementary tables of analyses on the first data set (N =905)**

**2-dimensional model**

|  | CEI | FI |
| --- | --- | --- |
| CCAS1 | 0.757 |  |
| CCAS2 | 0.754 |  |
| CCAS3 | 0.652 |  |
| CCAS4 | 0.695 |  |
| CCAS5 | 0.660 |  |
| CCAS6 | 0.782 |  |
| CCAS7 | 0.436 |  |
| CCAS8 | 0.600 |  |
| CCAS9 |  | 0.828 |
| CCAS10 |  | 0.620 |
| CCAS11 |  | 0.859 |
| CCAS12 |  | 0.844 |
| CCAS13 |  | 0.655 |

|  | CEI | FI |
| --- | --- | --- |
| Explained variance | 0.281 | 0.227 |

Factor correlation

|  | CEI | FI |
| --- | --- | --- |
| CEI | 1 |  |
| FI | 0.834 | 1 |

*Note*. CEI: cognitive-emotional impairment, FI: functional impairment

**Unidimensional item: N =905**

|  | F1 |
| --- | --- |
| CCAS1 | 0.727 |
| CCAS2 | 0.700 |
| CCAS3 | 0.579 |
| CCAS4 | 0.631 |
| CCAS5 | 0.635 |
| CCAS6 | 0.766 |
| CCAS7 | 0.445 |
| CCAS8 | 0.581 |
| CCAS9 | 0.790 |
| CCAS10 | 0.597 |
| CCAS11 | 0.811 |
| CCAS12 | 0.803 |
| CCAS13 | 0.651 |

|  | Eco-anxiety |
| --- | --- |
| Explained variance | 0.460 |


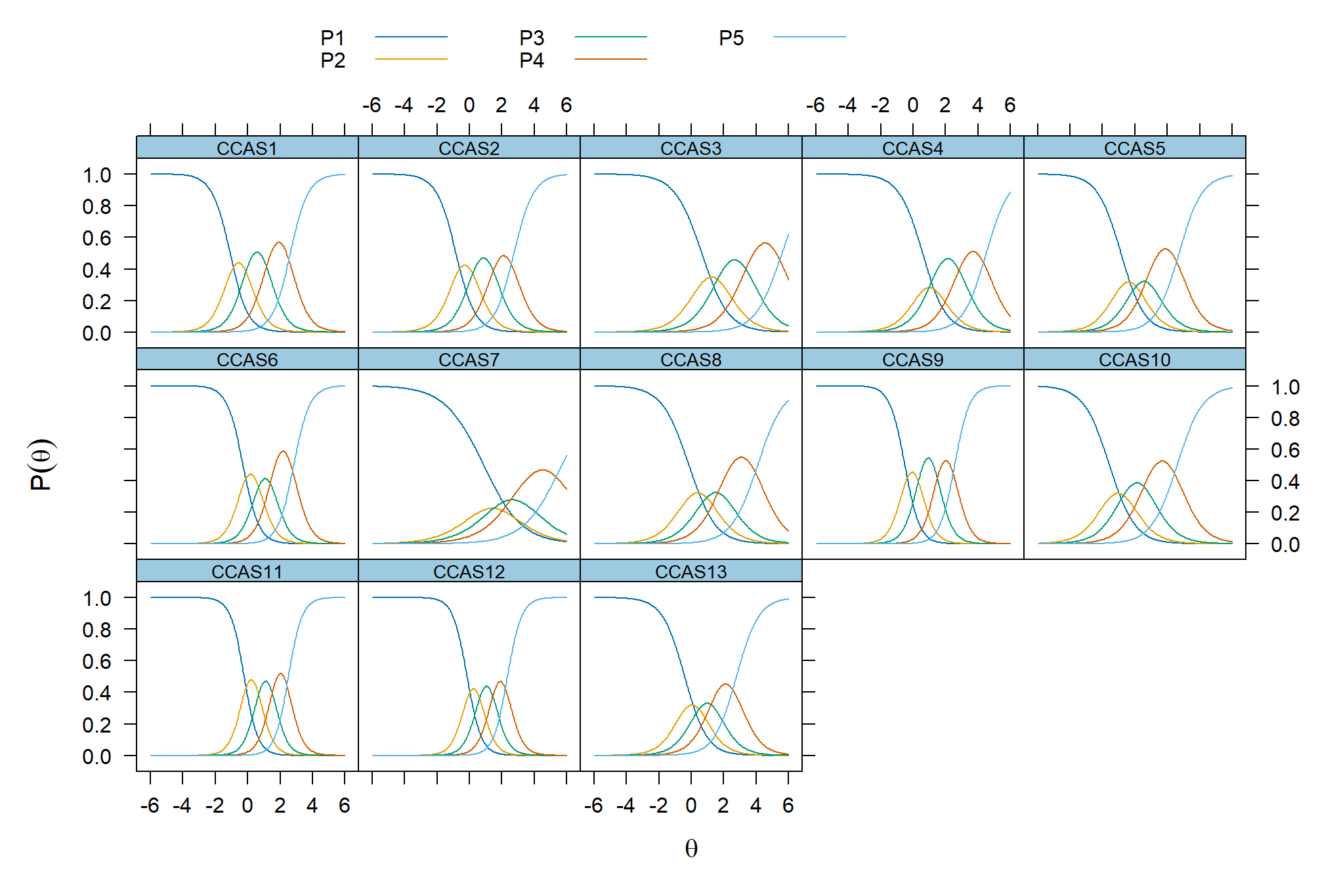


Item function curve: P1 to P5: item characteristic curve for the scale categories. P1 for “never”, P2 for “rarely”, P3 for “sometimes, P4 for “often”, P5 for “always”; P(θ): probability of selecting a category


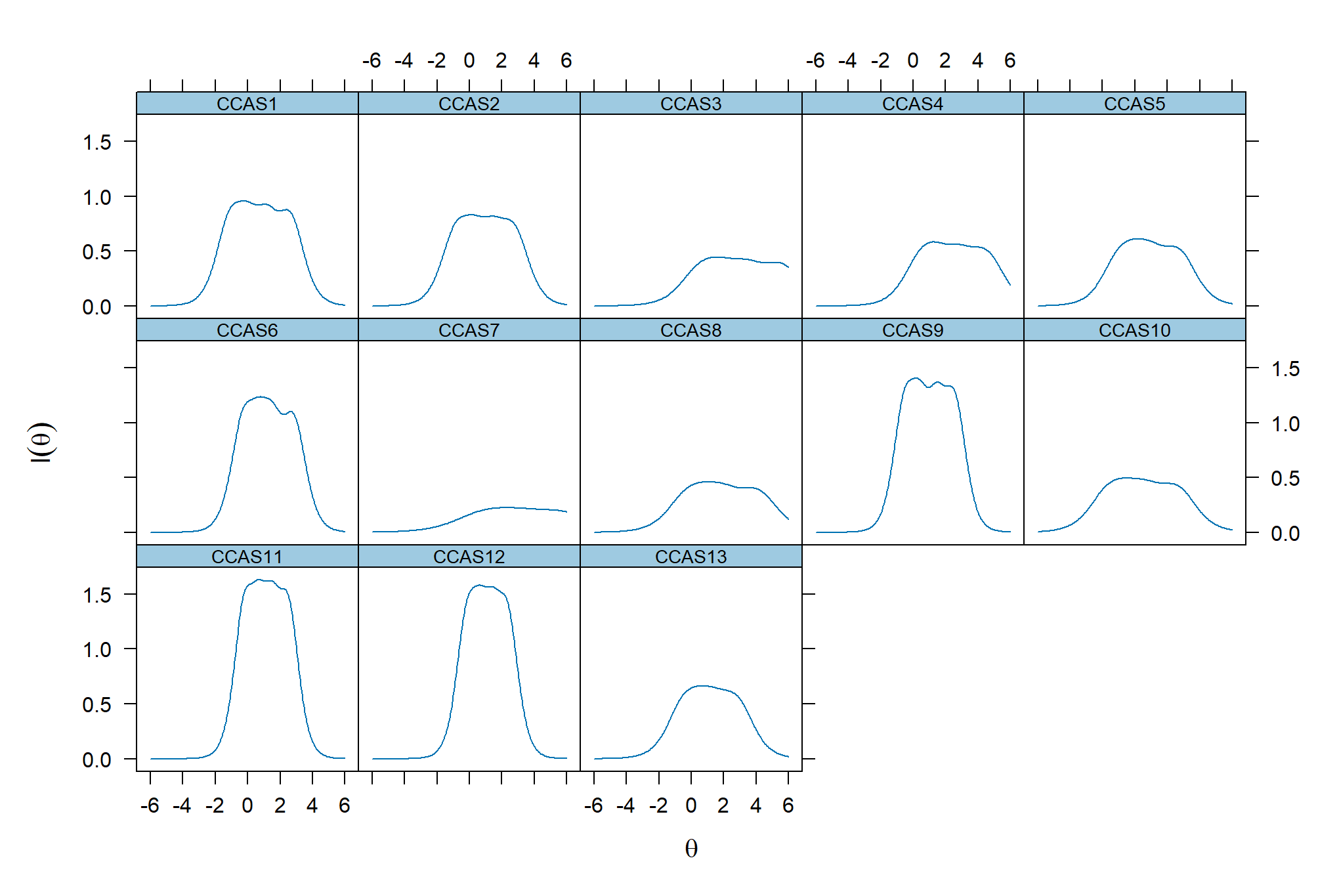


Item information curve (N= 905): I(θ) quantity of information about the latent trait provided by the item
